# Bioactive Sr(II)/Chitosan/Poly(ε-caprolactone) Scaffolds for Craniofacial Tissue Regeneration. In Vitro and In Vivo Behavior

**DOI:** 10.3390/polym10030279

**Published:** 2018-03-07

**Authors:** Itzia Rodríguez-Méndez, Mar Fernández-Gutiérrez, Amairany Rodríguez-Navarrete, Raúl Rosales-Ibáñez, Lorena Benito-Garzón, Blanca Vázquez-Lasa, Julio San Román

**Affiliations:** 1 Faculty of Chemistry, Autonomous University of San Luis Potosi, San Luis Potosi 6, Salvador Nava Martínez, 78210 San Luis, S.L.P., Mexico; itziamdz@gmail.com; 2 Institute of Polymer Science and Technology, ICTP-CSIC, C/Juan de la Cierva 3, 28006 Madrid, Spain; ictf339@ictp.csic.es (M.F.-G.); jsroman@ictp.csic.es (J.S.R.); 3 CIBER, Carlos III Health Institute, C/Monforte de Lemos 3-5, Pabellón 11, 28029 Madrid, Spain; 4 Faculty of Higher Studies, National Autonomous University of Mexico, Av. Chalma s/n Col. La Pastora, Cuautepec Barrio Bajo. Delegación Gustavo A. Madero, Ciudad de México 07160, Mexico; cd.anyrn@outlook.com (A.R.-N.); dr.raul.rosales@gmail.com (R.R.-I.); 5 Faculty of Medicine, University of Salamanca, C/Alfonso X el Sabio, s/n, 37007 Salamanca, Spain; lorenabenito@usal.es

**Keywords:** chitosan, PCL, strontium, scaffolds, craniofacial engineering

## Abstract

In craniofacial tissue regeneration, the current gold standard treatment is autologous bone grafting, however, it presents some disadvantages. Although new alternatives have emerged there is still an urgent demand of biodegradable scaffolds to act as extracellular matrix in the regeneration process. A potentially useful element in bone regeneration is strontium. It is known to promote stimulation of osteoblasts while inhibiting osteoclasts resorption, leading to neoformed bone. The present paper reports the preparation and characterization of strontium (Sr) containing hybrid scaffolds formed by a matrix of ionically cross-linked chitosan and microparticles of poly(ε-caprolactone) (PCL). These scaffolds of relatively facile fabrication were seeded with osteoblast-like cells (MG-63) and human bone marrow mesenchymal stem cells (hBMSCs) for application in craniofacial tissue regeneration. Membrane scaffolds were prepared using chitosan:PCL ratios of 1:2 and 1:1 and 5 wt % Sr salts. Characterization was performed addressing physico-chemical properties, swelling behavior, in vitro biological performance and in vivo biocompatibility. Overall, the composition, microstructure and swelling degree (≈245%) of scaffolds combine with the adequate dimensional stability, lack of toxicity, osteogenic activity in MG-63 cells and hBMSCs, along with the in vivo biocompatibility in rats allow considering this system as a promising biomaterial for the treatment of craniofacial tissue regeneration.

## 1. Background

Reconstruction of large bone defects still continue as a major challenge for orthopedists, and craniomaxillofacial surgeons. The craniofacial hard tissues work as a functional unit and provide structural support, protection, sensation and allow movement. Defect and dysfunction of bone can result in devastating deficits of bone in the craniofacial skeleton [1]. Total or partial loss of bone has many psychological and behavioral problems associated with facial deformities [2]. The repair of complex craniofacial bone defects is challenging [3] and a successful result mainly lies in the choice of reconstructive method [4].

Bone tissue engineering approaches have been developed as an alternative to conventional use of autologous bone grafts, allografts or demineralized bone matrix from a donor tissue into the patient. Bone substitutes are formed by a biomaterial scaffold that acts as mimetic extracellular matrix (ECM) to induce new functional bone regeneration. The scaffolds usually loaded with osteoconductive/osteoinductive components and stem cells [5,6] are intrinsically biocompatible and some of them have reached clinical use with minimal adverse immunological reports [5]. Osteoinductive components such as bioactive glasses [7,8], phosphate-based glasses [9] or hydroxyapatite (HAp) [10] have been investigated. In addition, strontium (Sr), zinc (Zn), magnesium (Mg) or copper (Cu) have been used to dope or modified biomaterials [7]. Particularly, Sr(II) is known to play an important role in promoting bone formation and osteoblasts stimulation while inhibiting osteoclasts resorption [11]. However, in clinical practice the medication of Sr(II) salts has been restricted after the secondary problems associated with the systemic administration. Thus, the application of systems based on a local delivery of the Sr(II) derivatives can be considered as an adequate strategy in order to take advantage of the excellent properties of Sr(II) avoiding the secondary problems. Consequently, different biomaterials containing Sr(II) ions have been prepared in recent years, some of them based on ceramics [7,8,9,12,13] or composites of synthetic polymers [14,15,16].

It is clear that the latest trends in the preparation of constructs for bone tissue engineering, especially craniofacial repair, are directed towards the use of biomaterials scaffolds that accommodate stem cells [17]. Abundant literature has been reported using mesenchymal stem cells (MSCs) [18,19] for this application [20,21,22,23,24,25] as well as using human dental pulp stem cells (hDPSCs) [26] and adipose tissue derived mesenchymal stem cells [19].

Bone substitutes for craniofacial bone repair can be made of natural and/or synthetic polymers [27,28], calcium phosphate ceramics [29], metals [30,31,32] and composites [33,34]. Different biomaterials have been employed to mimic ECM in cleft palate reconstruction [35,36]. Resorbable bioactive systems based on synthetic polylactic acid (PLA), poly(lactic-*co*-glycolic acid) (PLGA), poly(ε-caprolactone) (PCL) or natural collagen have been employed as barrier membranes for guided bone regeneration (GBR) in oral and maxillofacial reconstruction [37]. A revision focused on therapy methods, growth factors and scaffolds in alveolar cleft defects has been published by Khojasteh et al. [38].

Natural polymers offer the advantage of good biocompatibility and are bioactive as they can interact with the host tissues [39]. Among natural polymers, chitin [40,41,42,43] and chitosan are excellent candidates [44,45]. Recently Anitha et al. published a review on their applications including a discussion about the chitinous scaffolds obtained from marine sponges [46]. Chitosan, the deacetylated form of chitin, offers some advantages [45,47,48,49] which extend its capabilities in the field [46,50]. Its disadvantages are the weak mechanical properties and high rate of degradability. Thus, it is usually cross-linked and/or combined with other natural/synthetic polymers (e.g., blends) or used in composites. Chitosan has ability to promote proliferation and mineral matrix deposition by osteoblasts in culture [51] and it allows osteoconduction [52]. Enhanced osteoconductive properties and osteoinductive behavior can be achieved using composite scaffolds with ceramics [53] and incorporating growth factors; all this provides osteogenic response [54,55]. Thus, the use of chitosan in orthopedic/periodontal applications [46,49,56] and craniofacial bone defects repair [37,57] has increased over the years as well as the development of hybrid systems [58,59,60,61]. As mentioned above, in order to increase bioactivity many chitosan composites have been developed [44,62]. These composites enhance the osteogenic potential of the calcium compounds at the time that the polymer matrix inhibits migration of calcium compounds [63]. A variety of chitosan composites have been tested in vitro or in vivo for bone and craniofacial regeneration [44,64,65]. Vaca-Cornejo et al. evaluated the effects of chitosan in combination with HAp to promote alveolar bone growth in patients with periodontitis. After twelve months of the therapeutic strategy the chitosan/HAp implant reduced the pocket depth of the supporting tissue, the grading of tooth mobility and promoted alveolar bone growth; the patients conserved the dental organ, favoring a better quality of life [66]. Composite membranes formed by chitosan/alginate polymers and octacalcium phosphate/bioactive glasses were suitable for adhesion and growth of human bone marrow mesenchymal stem cells (hBMSCs) [67]. In 2017, Zhou et al. evaluated whitlockite (WH)/chitosan composite membranes and HAp/chitosan scaffolds, and they concluded that WH/chitosan scaffold can significantly promote bone regeneration in calvarial defects [68]. Recently, Lu et al. published that high-activity chitosan/nano HAp (nHAp)/zoledronic acid scaffolds had a multifunction of tumor therapy, bone repair, and antibacterial [69]. Even though numerous strategies that are currently used to regenerate bone depend on employing biocompatible materials exhibiting a scaffold structure. In this sense, Guzmán et al. have immobilized calcium phosphate salts and/or bone morphogenetic protein 2 (BMP-2) into chitosan scaffolds. Interestingly, they found that this multicomponent scaffold exhibited a superior efficacy in bone regeneration than the scaffolds containing only one of the components, either calcium phosphate salts or rhBMP2, separately in maxillary sinus augmentation procedure [70].

Numerous composite systems containing chitosan have found application for guided tissue regeneration (GTR) in periodontal tissue engineering. Some examples are: HAp–chitin–chitosan composite formulated as a self-hardening paste [71], membranes composed of electrospun chitosan fibers [72], two-layer nanofibrous membranes made of polyglycerol sebacate (PGS)/PCL/β-tricalcium phosphate (β-TCP) and PCL/PGS/chitosan that provide flexibility, osteoconductivity and barrier properties [73]. In vivo experiments with hybrid composite nanofibers composed of fish collagen/chitosan/bioactive glasses (BG) demonstrated bone regeneration in a furcation defect of dogs [74]. Tamburaci and Tihminlioglu mentioned that the incorporation of diatomite to chitosan polymer matrix significantly enhanced the osteoblast-like cell proliferation on membrane surface and can be used as an ideal candidate for GTR [75].

In addition, the preparation of chitosan based scaffolds doped with strontium has been addressed for bone tissue engineering and craniomaxillofacial repair and many of them are based on Sr-doped ceramics. Chitosan/strontium HAp (SrHAp) nanohybrid scaffolds with interconnected macropores and SrHAp nanocrystals produced favorable adhesion, spreading and proliferation of hBMSCs [10]. In addition, the Sr(II) ions released from the nanohybrid scaffolds enhanced alkaline phosphatase (ALP) activity and ECM mineralization [10]. Three-dimensional Ag-loaded SrHAp/chitosan porous scaffold also provided good support for the adhesion, spreading and proliferation of hBMSCs [76] showing that the Sr element increased the ALP activity, ECM mineralization, and the expression levels of osteogenic-related genes BMP-2 and collagen-I. Masaeli et al. studied the performance of a SrHAp additive in calcium phosphate cement. In vitro biological characteristics revealed that incorporation of 3 wt % SrHAp could cause ALP activity increase, which may be due to the presence of strontium ions [77]. Recently, our research group has developed semi-interpenetrating polymer networks (semi-IPNs) of biohybrid scaffolds composed of chitosan/polyethylene glycol dimethacrylate/β-TCP scaffolds loaded with a biocompatible strontium salt (i.e., strontium folate (SrFO) [78]). The scaffolds were seeded with stem cells obtained from hDPSCs to study the regeneration of bone using a critical sized defect model of calvaria in rats. The in vitro and in vivo results demonstrated excellent cytocompatibility with resorption of scaffolds in a period of 4–6 weeks and a total regeneration of the defect, with a more rapid and dense bone formation in the group with SrFO compared with unloaded scaffold [79].

The aim of this work focuses on the preparation of Sr(II) hybrid bioactive scaffolds applicable for regeneration of craniofacial defects. The designed scaffolds intend to cover the actual niche in clinical practice. The rational is to produce a biomaterial that is able of regenerating good quality of bone in the short or medium terms using biomaterials that are clinically employed in biomedical devices, i.e., chitosan [80] and PCL [81], to favor their translation to the commercial and clinical fields. In addition, the scaffolds contain Sr(II) as an osteogenic compound. Up to our knowledge, scaffolds of this composition are not reported yet in literature.

Thus, the paper describes the fabrication of Sr(II) impregnated chitosan/PCL hybrid scaffolds by a relatively simple two-steps method, aiming the Sr(II) availability for regeneration processes. Their morphology, structural characterization, physicochemical properties as well as swelling behavior are analyzed. Likewise, in vitro cytotoxicity and biological performance studying their osteogenic response are evaluated using osteoblast-like cells (MG-63) and hBMSCs. Finally in vivo biocompatibility experiments are carried out applying a subcutaneous pocket rat model.

## 2. Experiment

### 2.1. Materials

Chitosan (Ch) with degree of acetylation DA = 15% and intrinsic viscosity = 457 mL/G (25 °C, 0.1 M AcOH + 0.2 M NaCl) was gently provided by IDEBIO S.L. (Salamanca, Spain). Pharmaceutical grade chitosan with DA = 10% and *M*_w_ = 300 kDa purchased from Altakitin (Aveiro, Portugal) was used for biological and in vivo experiments. Poly(ε-caprolactone) (PCL, *M*_w_ = 14 kDa, Sigma-Aldrich, Madrid, Spain), sodium tripolyphosphate (TPP, 85% Sigma-Aldrich, Madrid, Spain), strontium fluoride (SrF_2_, Sigma-Aldrich, Madrid, Spain), 1,2-dichlorometane (DCM, Sigma-Aldrich, Madrid, Spain) and phosphate buffered saline solution (PBS) (pH = 7, Scharlau, Barcelona, Spain) were used as received.

### 2.2. Preparation of Scaffolds

2D membrane scaffolds with Ch:PCL ratios (*wt*/*wt*) of 1:2 and 1:1 were obtained by a casting/solvent evaporation technique. Briefly, in the first step chitosan (1 wt %) was dissolved in an aqueous solution of glacial acetic acid (0.25 wt %); separately, the PCL was dissolved in DCM and added to the chitosan solution under stirring. The final mixture was poured onto a Teflon mold, and dried at room temperature until constant weight. Then, dried membranes were cross-linked by dipping in a TPP (20 wt % respect to chitosan) aqueous solution for 24 h at room temperature. Then, membranes were removed from the solution, washed with 50 mM NaCl solution and distilled water after neutral pH, and dried until constant weight. In the second step, cross-linked membranes were treated by a drop-by-drop deposition of the SrF_2_ (5 wt % respect to chitosan) aqueous solution until complete wetting and left at room temperature for evaporation of solvent. Afterwards, treated membranes were washed with distilled water and dried at room temperature. The codes and compositions of the membrane scaffolds are shown in Table 1.

### 2.3. Characterization Techniques

Structural characterization was performed by attenuated total internal reflectance Fourier transform infrared (ATR-FTIR) spectroscopy with a Spectrum One apparatus (Perkin-Elmer, Madrid, Spain) spectrometer equipped with an ATR accessory.

Atomic composition of membranes was determined using a FE-SEM (Field emission scanning electron microscope, Tokyo, Japan) Hitachi SU-8000 with an energy dispersive X-rays (EDS) analyzer Bruker XFlash model Detector 5030 using a voltage of 8 keV.

Morphology of membranes was examined by scanning electron microscopy (SEM, Eindhoven, Hollad) using a Philips XL 30 microscope at an accelerating voltage of 25 kV. Thermal properties were analyzed by thermogravimetry (TGA) in a thermogravimetric analyzer TGA Q500 (TA Instruments, Cerdanyola del Vallés, Spain) apparatus, with a heating rate of 10 °C/min in a range of 40–500 °C and under nitrogen atmosphere (10 mL/min).

### 2.4. In Vitro Swelling Study

Swelling experiments were performed in PBS buffer (pH = 7) at 37 °C. Each sample was immersed in 5 mL of the medium and left to attain equilibrium under static conditions. The medium was replaced every 2 days. The percentage of the water uptake (*WU*) was calculated by Equation (1), where *W_t_* is the weight of the sample at time *t* and *W_d_* is the initial dry weight. Swelling measurements were performed at 1, 2, 7, 15, 30 and 45 days after immersion.

% *WU* = [(*W_t_* − *W_d_*)/*W_d_*] × 100 (1)

In all the experiments, a minimum of four replicates of each composition were measured and results averaged. Results are given as mean ± standard deviation (sd).

Additionally, to evaluate changes on the surface topology samples soaked for 30 days were washed with distilled water and dried for SEM analysis.

### 2.5. In Vitro Biological Assays

#### 2.5.1. Cell Cultures

MG-63 osteoblast-like cell line (ECACC, Sigma, Madrid, Spain) and human bone marrow mesenchymal stem cells, hBMSCs (Innoprot, Vizcaya, Spain), were used to study the biological performance of membranes scaffolds. The culture medium for MG-63 line was Dulbecco’s modified Eagle’s medium enriched with 4500 mg/mL glucose (DMEM) (Sigma, Madrid, Spain) supplemented with 10% fetal bovine serum (FBS), 200 mM l-glutamine, 100 units/mL penicillin and 100 μg/mL streptomycin, and modified with HEPES (complete medium). In the case of hBMSCs the culture medium was basal medium supplemented with 5% of FBS, 5 mL of mesenchymal stem cell growth supplement (MSCGS), 100 units/mL penicillin and 100 μg/mL streptomycin (Innoprot, Vizcaya, Spain). Thermanox^®^ (TMX) discs (Nunc) were used as negative control. Tested sample membranes (1.5 cm diameter) were sterilized with a UV lamp (HNS Osram, 263 nm, 3.6 UVC/W) at a power of 11 W for 4 h.

#### 2.5.2. Biological Assays

MTT test [82] was used for indirect cytotoxicity. Tested samples were set up in 5 mL of FBS-free supplemented DMEM, placed on a shaker at 37 °C and extracts were obtained at 1, 2, 7, 14 and 21 days under sterile conditions. Cells were seeded at a density of 9 × 10^4^ cells/mL in complete medium in a sterile 96-well culture plate and incubated to confluence. After 24 h incubation the medium was replaced with the corresponding extract and incubated at 37 °C in humidified air with 5% CO_2_ for 24 h. A solution of MTT (0.5 mg/mL) was prepared in warm FBS-free supplemented DMEM and the plates were incubated at 37 °C for 3–4 h. Excess medium and MTT were removed and DMSO was added to all wells in order to dissolve the MTT taken up by the cells. This was mixed for 10 min and the absorbance was measured with a Biotek Synergy HT detector using a test wavelength of 570 nm and a reference wavelength of 630 nm. Relative cell viability was calculated from Equation (2):

Relative cell viability (%) = 100 × (*OD_S_* − *OD_B_*)/(*OD_C_* − *OD_B_*) (2)

where *OD_S_*, *OD_B_* and *OD_C_* are the optical density for the sample (S), blank (B) and control (C), respectively. Results are given as mean ± standard deviation (sd) (*n* = 5). Analysis of variance (ANOVA) of the results was performed comparing samples with TMX (* *p* < 0.05).

Quantitative analysis for cell adhesion and proliferation on membrane scaffolds was carried out by means of the Alamar Blue (AB) test [83]. Cells were seeded at a density of 4 × 10^4^ cell/mL for 24 h over the specimens in a 24-well culture plate. At determinate times (1, 4, 7, 14 and 21 days), 1 mL of AB dye (10% AB solution in phenol red free DMEM medium) was added to each specimen. After 4 h of incubation 100 µL (*n* = 4) of culture medium for each test sample was transferred to a 96-well plate, and the fluorescence emission was measured at 590 nm in a Biotek Synergy HT. The specimens were washed twice with PBS to remove rest of the reagent, and 1 mL of culture medium was added to monitor the cells over the materials. Results are given as mean ± sd. ANOVA of the results of tested materials was performed comparing the corresponding Sr(II) and blank groups at the same time (* *p* < 0.05).

Total DNA was measured using the PicoGreen dsDNA quantitation kit (P-7589, Molecular Probes, Fisher Scientific, Madrid, Spain). The recently introduced fluorescent dye, PicoGreen, has several advantages over other methods because it is sensitive and specific for double-stranded DNA (dsDNA) [84].

Biochemical detection of ALP activity was used as an indicator of osteoblast phenotype [85]. The ALP activity was evaluated in confluent cells cultured in the presence of tested sample. ALP catalyzes the hydrolysis of *p*-nitrophenyl phosphate (pNPP) to *p*-nitrophenol. It has a strong absorbance at 405 nm. The rate of the increased absorbance at 405 nm is proportional to the enzyme activity. Determination of the ALP/DNA ratio is indicative of the amount of ALP activity per cell. The variations caused by the different shape of the test samples can be eliminated using this approach [86]. Both were measured from cell lysate. Results are given as mean ± sd. ANOVA of the results of tested materials was performed comparing the corresponding Sr(II) and blank groups at the same time (* *p* < 0.05).

Cell morphology was examined by SEM. To that end, samples were placed in a 24-well tissue-culture plate. Cells (4 × 10^4^ cells per well) were added and allowed to attach at 37 °C. Samples were washed three times with distilled sterile water and fixed with 2.5% glutaraldehyde for 2 h at room temperature. The dried samples were mounted on aluminum stumps and sputter-coated with gold/palladium mix before examination under a SEM apparatus (Philips XL 30) at an accelerating voltage of 15 kV.

### 2.6. In Vivo Biocompatibility

#### 2.6.1. Animal Experimentation

All animal studies were performed according to the national guidelines and conducted in accordance with Spanish law (RD 53/2013) and international standards on animal welfare as defined by European Directive (2010/63/EU). In addition, surgery protocols were approved by the Ethical Committee (Project Identification Ethical Committee: 211, 17 November 2017) of the University of Salamanca, Salamanca, Spain. The animals were housed in cages with pelleted food and water in a temperature-controlled room with a 12 h artificial day/night cycle at the Animal Experimentation Unit (A.E.U.) of the University of Salamanca. They were acclimatized far at least 2 weeks prior surgery.

#### 2.6.2. Subcutaneous Implantation in Rats

The biocompatibility of the membranes was assessed in the subcutaneous of rats. Twenty-one albino Wistar male rats, body weight 250–300 g, were purchased from a certificated stockbreeder (Charles River, Barcelona, Spain). The rats were placed under general anesthesia by inhalation of 1.5% isofluorane (Forane^®^). Pre- and post-operative analgesia was provided by subcutaneous injection of buprenorphine (0.01–0.05 mg/kg). The back of each rat was depilated on using an electric shaver and disinfected with povidone iodine 10% solution (Betadine^®^). A sterile field was placed on the back of the animal. Incisions were made through the skin on each side to the midline, along the vertebral column, to made unconnected subcutaneous pouches for each sample (three incisions in total) with 2 cm distance from each other. Three unconnected subcutaneous pockets were created by means of blunt dissection. Each rat received 3 membranes (1 × 1 cm^2^), each one in a separate subcutaneous pocket: control membrane of bovine collagen (RCM6, ACE Surgical Supply Inc., Brockton, MA, USA) and selected studied membranes: Ch/PCL and Sr/Ch/PLC. After membrane implantation skin was sutured using non-absorbable 4/0 silk suture (Aragó, Barcelona, Spain). At each time period, 1, 2 and 4 weeks of implantation, animals (*n* = 7) were euthanized by lethal injection of 5% sodium pentobarbital (Dolethal^®^) and implants retrieved for histological evaluation. After dissection, samples isolating each membrane were fixed in 4% neutral formaldehyde.

#### 2.6.3. Histological Analysis

Once fixed, samples were placed in cassettes and dehydrated in ascending series of 70%, 80%, 90% and 100% ethanol solutions. Then, they were placed into ethanol/toluene and pure toluene before being immersed in liquid paraffin at 60 °C. Afterwards, samples were embedded in paraffin blocks at −20 °C. Blocks were cut by using a standard rotatory microtome (Micron HM310, Walldorf, Germany). Thin histology sections (5 µm) were made perpendicular to the plane of the skin and stained with hematoxylin and eosin (H-E).

Sections were microscopically blinded viewed to determine the histological reaction to the membranes, specifically the presence and degree of inflammatory cell response. Sections were also examined to observe fibrous tissue and vascularization.

## 3. Results

### 3.1. Preparation and Characterization of Membrane Scaffolds

Biohybrid Sr(II) containing Ch/PCL membranes were fabricated using a two-step way methodology as it is described in the experimental section. Chemical composition of the blank samples was analyzed by ATR-FTIR spectroscopy. ATR-FTIR spectra (Figure S1) presented the characteristic bands belonging to pure precursor polymers but with some differences. In the hybrid scaffolds the band between 3500 and 3200 cm^−1^ (associated υ O–H and υ N–H) broadened compared to pure chitosan. In the region between 1750–1500 cm^−1^, the band at 1722 cm^−1^ for Ch/2PCL and 1723 cm^−1^ for Ch/PCL (υ C=O in ester groups belonging to PCL) shifted with respect to pure PCL (1721 cm^−1^); the bands at 1656 cm^−1^ for Ch/2PCL and 1646 cm^−1^ for Ch/PCL (υ C=O in amide groups, amide I of chitosan) shifted compared to pure polymer (1654 cm^−1^), mainly for the Ch/2PCL sample; the bands at 1587 cm^−1^ for Ch/2PCL and 1586 cm^−1^ for Ch/PCL (δ N–H in NH_3_^+^ groups) also appeared shifted respect to those of pure chitosan (1578 cm^−1^). On the other hand, bands in the region between 1300–900 cm^−1^ were assigned to υ_as_ PO_2_ groups in TPP ions [87], υ C–O in the pyranose ring of chitosan [88] and υ_s_ and υ_as_ C–O in PCL [89].

**Figure 1:**
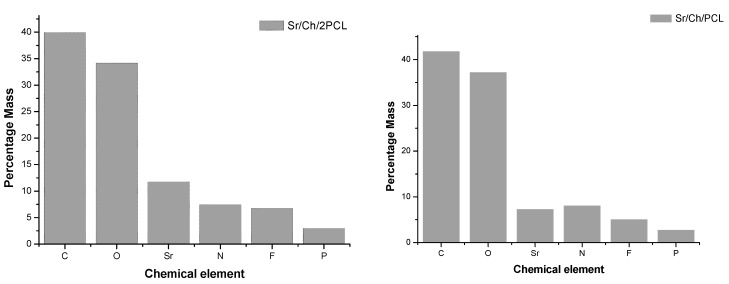
EDS chemical element percent mass of Sr/Ch/2PCL and Sr/Ch/PCL dried samples. Ch: chitosan; PCL: poly(ε-caprolactone).

The atomic composition of dried Sr(II) samples was examined by EDS analysis. For both sample compositions EDS spectra exhibited the peaks of C and O pertaining to both PCL and chitosan polymers, the peak of N belonging exclusively to the polysaccharide and the peak of Sr centered in 1.8 keV, indicating the presence of the bioactive Sr(II). In addition, a peak centered at 2 keV (P) belonging to TPP polyanions was appreciated [90]. Figure 1 shows the EDS results for the main elements of Sr(II) samples. Although this analysis must be considered semi-quantitative, a higher amount of Sr was measured in the sample with higher content of PCL.

**Figure 2:**
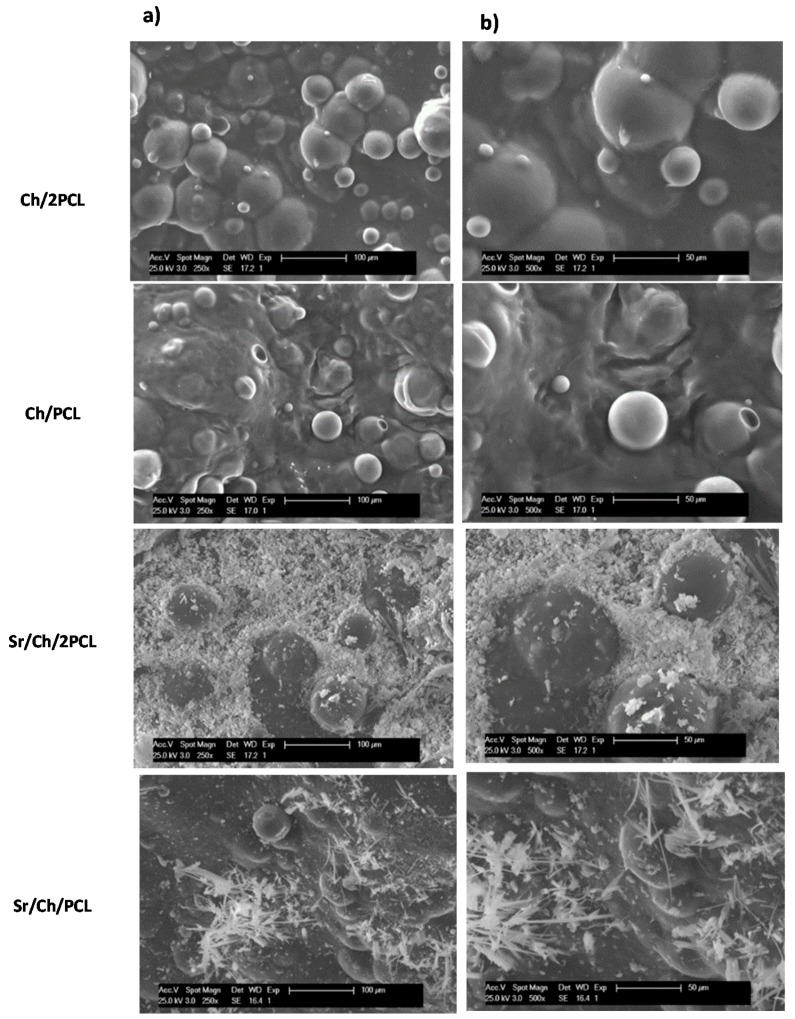
SEM images of dried blank and Sr(II) samples. (**a**) 250×; (**b**) 500×. Ch: chitosan; PCL: poly(ε-caprolactone).

Surface morphology of membranes with and without Sr(II) was analyzed by SEM and images are displayed in Figure 2. It is clear that all membranes presented phase separation morphology consisted of PCL microparticles dispersed in a continuous matrix of cross-linked chitosan. As far as Sr(II) membranes is concerned, the Sr salt was preferably located on the hydrophilic polysaccharide matrix. It seems that the hydrophobic character of PCL in water does not favor the diffusion of the Sr salt solution into the microparticles. For Ch:PCL 1:2 ratio, the higher concentration of PCL microparticles makes the chitosan phase more concentrated in the Sr salt than for the 1:1 ratio.

### 3.2. Thermal Properties

**Figure 3:**
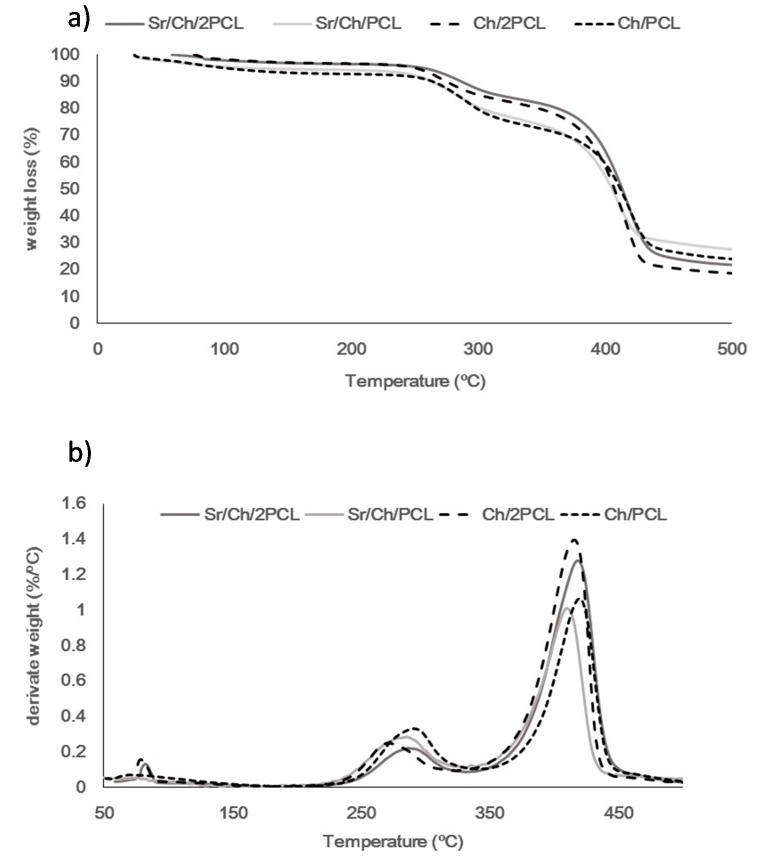
TGA (Termogravimetric analysis) (**a**) and DTG (Derivative Thermogravimetric analysis) curves (**b**) of blank and Sr(II) membranes under nitrogen atmosphere. Ch: chitosan; PCL: poly(ε-caprolactone).

Thermograms and Derivative Thermogravimetric analysis (DTG) of membranes obtained under nitrogen atmosphere are shown in Figure 3. Thermogravimetric results of both pure PCL and chitosan are shown in Figure S2.

Thermal degradation of PCL occurred in two steps of which DTG curve showed a *T*_max1_ at 307 °C and a *T*_max2_ at 419 °C. The first degradation step generates H_2_O, CO_2_, and 5-hexenoic acid as evolved products and the second one leads to the formation of ε-caprolactone (cyclic monomer) as a result of an unzipping depolymerization process [91]. TGA of chitosan showed a main degradation stage with maximum rate at 287 °C ascribed to deacetylation of the main chain and cleavage of their glycosidic linkages [88]. Blank and Sr(II) membranes thermally degraded in two steps, with *T*_max1_ and *T*_max2_ in DTG curves in the ranges 250–350 °C and 350–450 °C. The first step was ascribed to degradation of the chitosan matrix and part of PCL whereas the second one was only due to chain scission of the synthetic polymer. Table S1 shows the thermogravimetric results of all samples. There was little difference between blank and Sr(II) samples. In general, *T*_max1_ decreased compared to the average of those of both pure components. The drop was more market for the Ch/2PCL sample and it was closer to *T*_max1_ of the PCL.

### 3.3. Swelling Behaviour

**Figure 4:**
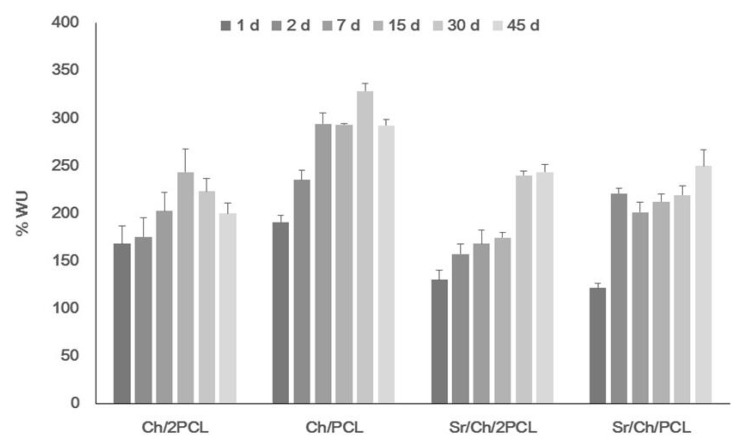
Variation of water uptake of membranes after immersion in PBS buffer at 37 °C. Ch: chitosan; PCL: poly(ε-caprolactone).

In vitro swelling of all samples was analyzed in PBS buffer at 37 °C. Results are represented in Figure 4. All samples exhibited complete swelling, very rapid within the first 24 h, and from then on, hydration progressively and slowly increased up to reach a maximum value. Ch/2PCL sample absorbed 167% of water at 1 day whereas Ch/PCL sample 190%, and maximum *WU* values were 243% and 328% respectively, with decreasing PCL content. After the maximum, *WU* slightly decreased to values of 200% and 300% respectively; this phenomenon can be attributed to some degradation of matrix by breakdown of some ionic cross-linking. Sr bioactive membrane scaffolds absorbed 130% (Sr/Ch/2PCL) and 121% (Sr/Ch/PCL) of water at one day of soaking and absorption progressed until, maximum *WU* values were around 245% for both samples at 45 days. Comparing blank and Sr(II) samples, maximum *WU* were higher for the blanks in all the studied period, what can be attributed to delivery of Sr in the latter samples. Additionally, changes in morphology surface under wet conditions for Sr(II) samples at 30 days were analyzed by SEM (Figure S3). Few differences were observed between samples of varying composition. Generally, Sr(II) crystals considerably decreased after 30 days immersion, and signs of chitosan matrix erosion were incipient but PCL microparticles remained unaltered at this time.

### 3.4. In Vitro Biological Behaviour

Cytotoxicity of samples was evaluated against MG-63 cell and hBMSCs with the MTT assay that measures the succinate mitochondrial dehydrogenase enzyme activity [82]. The results are shown in Figure S4; cell viability values in presence of lixiviates taken at 1, 2, 7, 14 and 21 days of all samples ranged around 100% reflecting absence of in vitro cytotoxicity according to standard specifications [92].

#### 3.4.1. Osteoblasts-Like Cells

**Figure 5:**
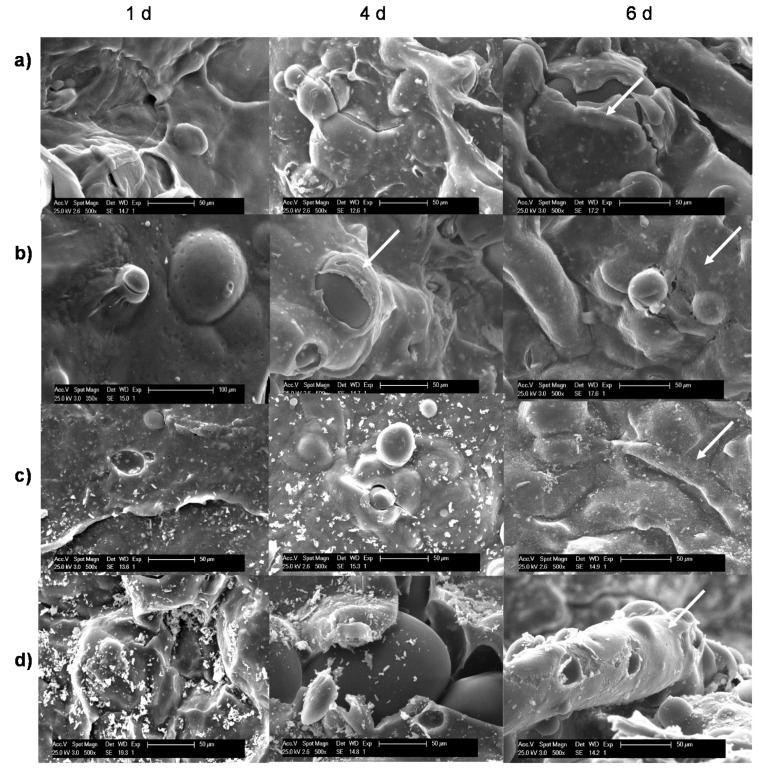
SEM images of MG-63 cells colonization on Sr(II) and blank membrane scaffolds at different post-seeding times. (**a**) Ch/2PCL; (**b**) Ch/PCL; (**c**) Sr/Ch/2PCL and (**d**) Sr/Ch/ PCL. Ch: chitosan; PCL: poly(ε-caprolactone).

SEM examination of blank and Sr(II) samples directly seeded with MG-63 cells was performed to study adhesion and cellular morphology. Figure 5 shows the SEM images of the osteoblast-like cells colonization on samples at different times. Cells showing adhesion and spread morphology are signalized by white arrows on the micrographs. Cells showed much better adhesion and spreading on the systems containing Sr(II) compared with those detected on blank samples. Interestingly, SEM examination revealed that qualitatively, cell growth and extracellular matrix formation was much higher on the Sr/Ch/PCL sample in the studied period but especially at six days. However, it is worth mentioning that blank samples also provided adhesion of cells with good spreading and adaptation to the surface although in a much lower extend.

**Figure 6:**
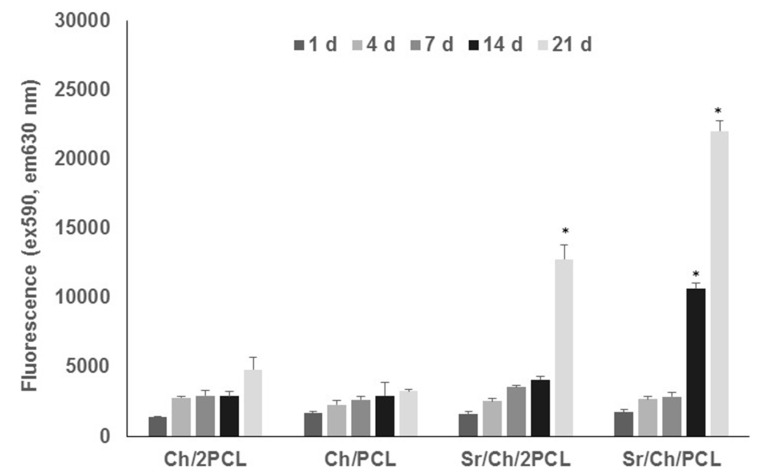
Alamar Blue results for blank and Sr(II) membranes in MG-63 cells over a period of 21 days. Results are given as mean ± sd (*n* = 5). Asterisks (*) indicate a significant difference comparing the corresponding Sr(II) and blank groups at the same time (* *p* < 0.05). Ch: chitosan; PCL: poly(ε-caprolactone).

To quantify cell proliferation AB assay [93] was carried out. The results of this test are shown in Figure 6. In the Sr(II) membranes the increase of fluorescence from 1 to 21 days indicated a higher number of viable cells over time. In particular, the Sr/Ch/2PCL formulation showed a significantly higher cell growth than its blank at 21 days, and the Sr/Ch/PCL sample showed same result at both 14 and 21 days. Blank membranes, on the other hand, behaved differently, and cell viability remained low in all studied times. Therefore, in the light of SEM and AB findings, the Sr(II) membrane with lower PCL content was selected for further cellular studies.

**Figure 7:**
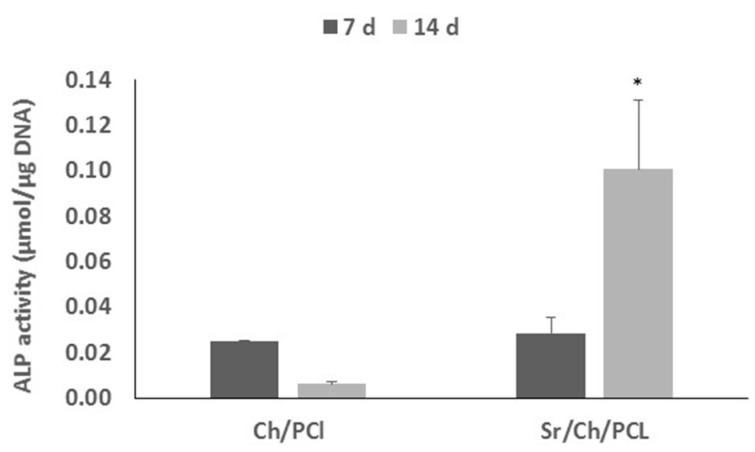
ALP/DNA activity in MG-63 cells cultured directly on test materials over a period of 14 days. Results are the mean ± sd (*n* = 5). Asterisks (*) indicate a significant difference comparing the two groups at the same time (* *p* < 0.05). Ch: chitosan; PCL: poly(ε-caprolactone).

Osteogenic response of Sr/Ch/PCL sample was evaluated and ALP levels were normalized for DNA. Results are plotted in Figure 7. A significant increase in ALP activity was observed for the Sr/Ch/PCL materials compared with the blank at 14 days culture period.

#### 3.4.2. Human Bone Marrow Mesenchymal Stem Cells

**Figure 8:**
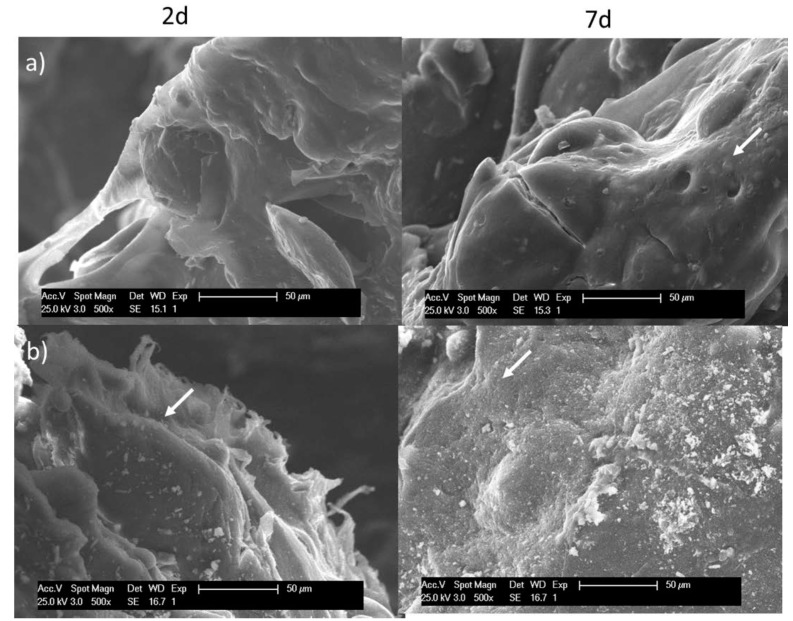
SEM images of hBMSCs colonization on blank and Sr(II) scaffolds at different times post-seeding. (**a**) Ch/PCL and (**b**) Sr/Ch/PCL. Ch: chitosan; PCL: poly(ε-caprolactone).

Blank and Sr/Ch/PCL membranes were seeded with hBMSCs and the morphology of cells grown was studied by SEM, after two and seven days (Figure 8). Cells adhesion and spread morphology are signalized by white arrows on the micrographs. In the case of membranes with Sr(II), good adhesion and proliferation at both studied times can be observed. We can also see that cells grew on the blank membranes but only at 7 days after seeding and in a lower extent.

**Figure 9:**
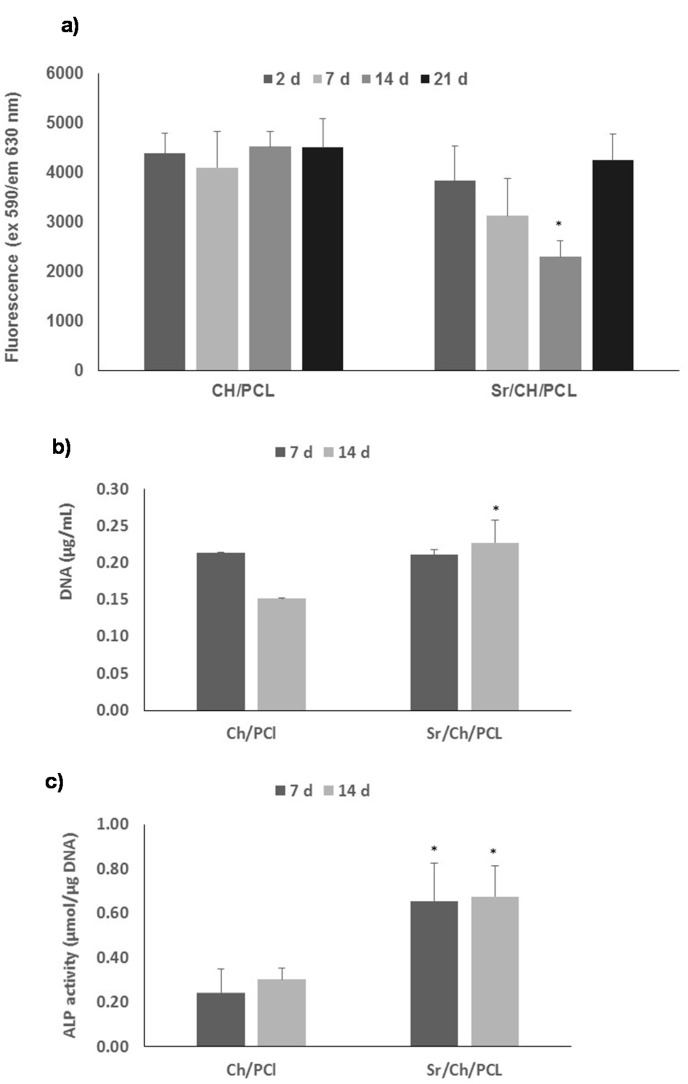
(**a**) Cell proliferation results obtained in AB assay of hBMSCs culture directly on test materials over a period of 21 days; (**b**) DNA (µg/mL) in cell lysate over a period of 14 days; (**c**) ALP/DNA activity over a period of 14 days. Results are the mean ± sd (*n* = 4). Asterisks (*) indicate a significant difference comparing blank and Sr(II) samples at the same time (* *p* < 0.05). Ch: chitosan; PCL: poly(ε-caprolactone).

AB assay results (Figure 9a) showed a significantly lower cell proliferation for the Sr(II) membranes compared with the blank at 14 days although cell viability recuperated giving no significant differences at 21 days. Examination of DNA content in the period between seven and 14 days (Figure 9b) indicated that DNA was significantly higher in Sr(II) membranes compared with the blank at 14 days and ALP content normalized for DNA (Figure 9c) showed a significantly higher ALP activity compared with the blank over the 14 days culture period.

### 3.5. In Vivo Biocompatibility

In vivo biocompatibility was studied in a subcutaneous model in rats in the system Sr/Ch/PCL and its blank using collagen membranes as control. Animals were sequentially sacrificed at each time point in post-operative days (7, 14 and 28 days). No behavioral changes or visible signs of physical impairment indicating systemic or neurological toxicity were observed between post-operative examination and the time of sacrifice. Macroscopic examination showed good wound closures without signs of inflammation. After 28 days all membranes were visible.

**Figure 10:**
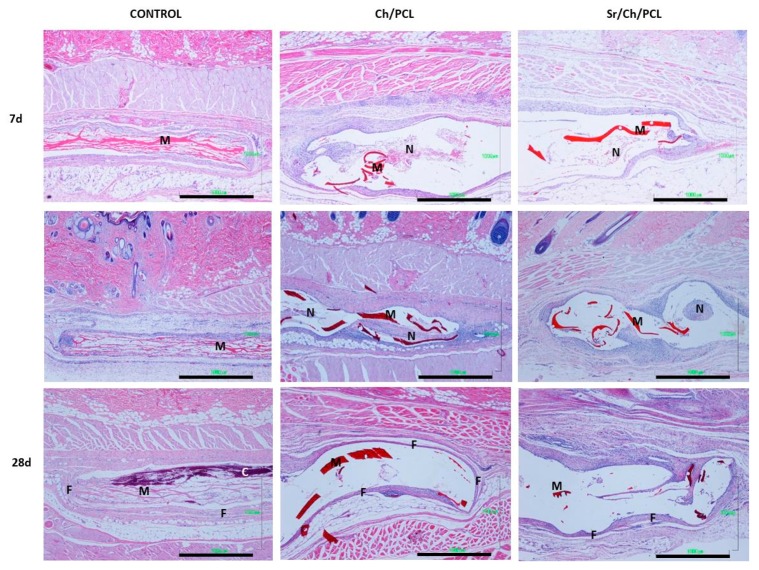
Micrographs of rat subcutaneous tissue responses to Control, Ch/PCL and Sr/Ch/PLC membranes after different implantation times (7, 14 and 28 days). M: membrane, N: necrotic tissue, F: fibrous tissue, C: calcification (H-E, 4×, scale bar 1 mm). Ch: chitosan; PCL: poly(ε-caprolactone).

**Figure 11:**
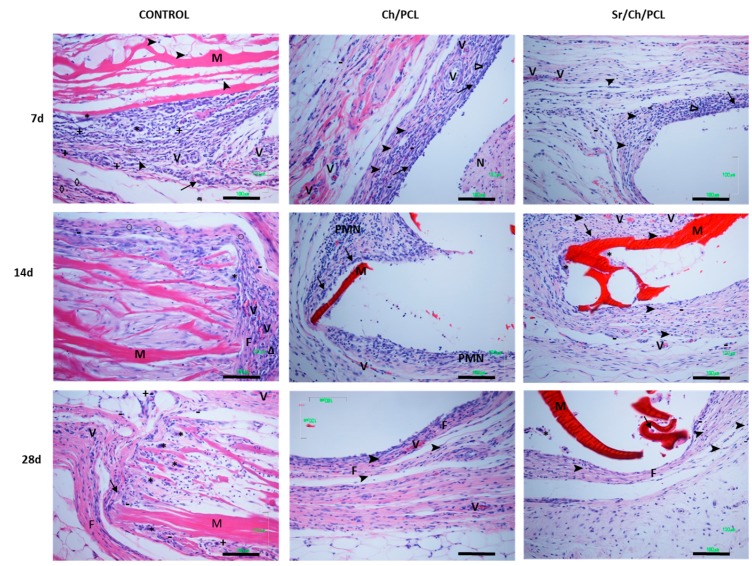
Micrographs of rat subcutaneous tissue responses to Control, Ch/PCL and Sr/Ch/PLC membranes after different implantation times (7, 14 and 28 days). M: membrane; N: necrotic tissue; F: fibrous tissue; V: blood vessels; →: macrophages; *: multinucleated cells; +: mast cells; **➤**: lymphocytes; ◊: eosinophils; **○**: plasmatic cells; −: monocytes; **∆**: leucocytes; PMN: polymorph nuclear cells. (H-E, 20×, scale bar 100 µm). Ch: chitosan; PCL: poly(ε-caprolactone).

Detail microscopic histology images of the control (collagen), Ch/PCL and Sr/Ch/PLC membranes at specific time points are shown in Figure 10 and Figure 11. The inflammatory responses of all membranes decreased with the time after implantation. At early experimentation time, typical inflammation repair process was developed composed by an infiltrate of inflammatory cells surrounded the membranes. However at 28 days of study, due to the resolution of the inflammatory (repair) process, less cellularity response was evident, where only few focal inflammatory reactions were evident.

The studied membrane specimens showed no degeneration of the structure but they were fragmented and showed cracks. However, control collagen membranes appeared unstructured forming like a mesh.

#### 3.5.1. Control Group

Histology images of control collagen membranes are shown in Figure 10 and Figure 11. After 7 days, slight acute inflammation was evident, characterized by the infiltration of mononuclear macrophages, mast cells, and eosinophils. It could be noticed the beginning of multinucleated cells formation. In the collagen membrane, there was little cell colonization, composed by polymorph nuclear cells and lymphocytes, particularly after short implantation time. The membrane was disorganized, forming a mesh-like structure. Focal development of small blood vessels was observed.

At 14 days, control collagen membranes appeared fragmented. Collagen membranes evoked a mild inflammatory reaction characterized by the infiltration of plasmatic cells, monocytes, leucocytes and eosinophils. Additionally, formation of multinucleated cells and local fibrous tissue were evident. An evidence of membrane degradation increased. Moderate focal blood vessels were developed.

Finally, at 28 days, control group evoked a mild local inflammatory reaction characterized by minimal infiltration of monocytes, macrophages, mast cells and scarce multinucleated cells. Fibroblasts presented activate synthesis of collagen fibers and formation of local fibrous tissue. Calcification process was evident in the collagen membranes remnants due to the augmented appetence to the basophilic stain (dark purple stain). An increased degradation and delamination of membrane material was observed. It could be observed less collagen fibers which were thinner and separated/spread during time.

#### 3.5.2. Ch/PLC

Histology images of Ch/PLC membranes (Figure 10 and Figure 11) after 7 days of subcutaneous implantation showed a typical acute inflammatory infiltrate with occasional areas of fibrinoid necrosis as well as some foci of foreign body reactions. At the membrane periphery the cellular infiltrated was characterized by macrophages and leucocytes. The surrounding tissue showed a mild inflammatory reaction composed of monocytes, macrophages, lymphocytes and few leucocytes. Moderate neovascularization was observed surrounded the membranes. Fibroblasts were presented but no fibrosis was evident.

At 14 days, the necrotic areas were reduced and vascularized, with infiltration composed mainly by macrophages and polymorphonuclears cells. Macrophages were active and phagocytosis processes were evident. A mild inflammation infiltrate was mainly composed of macrophages and leucocytes with progressive fibrosis.

After 28 days of implantation, mild focal inflammatory reaction with little vascularization was evident. The infiltrate was predominantly composed by macrophages and lymphocytes. Histological observations proved highly organized collagen fibers, demonstrating fibroblasts activity and focal fibrosis formation around membranes. Despite fibrous tissue proliferation no capsule formation occurred.

#### 3.5.3. Sr/Ch/PLC

The histological response induced by Sr/Ch/PLC membranes is displayed in Figure 10 and Figure 11. After 7 days of implantation, Sr/Ch/PLC evoked a mild local inflammatory reaction characterized by the infiltration of monocytes, macrophages and lymphocytes. Occasional small areas of necrosis were evident with an infiltrate of polymorphonuclear cells. At the membrane periphery the minor cellular infiltrated was characterized by leucocytes. No significant neovascularization was developed. Fibroblasts were present but no fibrosis was formed.

At 14 days, the necrotic areas almost disappeared and were vascularized allowing the cellular infiltration of macrophages and polymorphonuclear cells. The surrounding tissues showed signs of a slight focal inflammation at a very advanced stage of resolution, exhibiting good blood supply. The infiltrate was predominantly composed of monocytes, macrophages and lymphocytes. When small membrane fragments were present, formation of multinucleated cells occurred surrounding them. Minimal fibrous tissue proliferation was detected.

Finally, after 28 days, slight focal inflammatory reaction at an advanced stage of resolution with little vascularization was evident. The inflammatory infiltrate was characterized by the presence of monocytes, macrophages and lymphocytes. Also multinucleated cells were seen in association with small membrane fragments, if were present. Minimal fibrous tissue proliferation was detected composed by few collagen fibers parallel organized. Focal fibrosis formation occurred around membrane so no capsule formation happened.

## 4. Discussion

The main goal of this work deals with the preparation of bioactive hybrid scaffolds in respond to the clinical demand of bioactive systems intended for craniofacial defects regeneration. The scaffolds studied are membranes of a chitosan and PCL hybrid system, finally doped with Sr(II). The biodegradable polysaccharide with ionizable groups in its chemical structure and highly hydrophilic character will endow swelling and biological properties [71] whereas the PCL, a semi-crystalline and biodegradable polymer with a hydrophobic character, will contribute to the biomechanical stability [81]. Strontium component, on the other hand, will confer osteogenic response. Thus, the Sr(II) membranes were fabricated using a two-steps procedure. In the first step, membranes were obtained by casting a mixture of polymers solutions to be later ionically cross-linked by an immersion process in a TPP solution [87,94,95]. It is worth saying that the mixture of the solution of PCL in DCM into the aqueous solution of chitosan gives rise to the formation of PCL microparticles dispersed in a continuous matrix of chitosan after evaporation of solvents. In the second step, the cross-linked membranes were impregnated with a Sr(II) salt solution what conducted to incorporation of Sr(II) in scaffold areas close to or on the surface. The preparative methodology intends to get interactions of Sr(II) ions with the hybrid system while maintaining strontium availability to the biological medium. Systems based on chitosan and PCL without strontium element are well documented in literature for general tissue engineering. Actually, some of them have recently developed. They can consist of blends [96,97], bilayer systems [98] and functionalized coatings [99]. Other systems have been prepared following nano [100], micro and macro approaches [101]. As far as our knowledge is concerned, systems containing Sr(II) composed of chitosan/PCL have not been reported yet. However, there are systems containing Sr(II) that are separately based on these polymers. They were fabricated following different approaches. Composite scaffolds of chitosan/polymethacrylates loaded with SrFO were obtained by free radical polymerization of macromonomers, giving rise to porous scaffolds in which SrFO was homogeneously distributed along the polymeric matrix [79]. Freeze-drying fabrication of Ch/SrHAp composite scaffolds were reported by Xu et al. [76] and Lei et al. [10]. Respect to PCL approaches, Ren et al. developed a PCL/Sr-substituted 45S5 Bioglass^®^ (SrBG) composite scaffold produced by melt electrospinning [102]. Other PCL/SrBG composite scaffolds were fabricated using an additive manufacturing technique. The bioactive particles were distributed in the PCL bulk across the scaffold (micro-CT examination) but SEM images revealed visible SrBG particles on their surface [16]. PCL and strontium porous composite scaffolds were fabricated using a simple one-step process to simultaneously foam PCL containing a strontium- and calcium carbonate. Integration of the inorganic educts into the scaffolds was observed by EDS-spectroscopy [15].

Morphology of membranes prepared in this work showed segregation of both components with microdomains of PCL microparticles distributed in a continuous matrix of the natural polymer as it has been commented above. This phenomenon can derive from both the structural characteristics of polymers and the methodology applied. On the one hand, the different hydrophilicicity of the synthetic and natural polymers contributes to the formation of a non-compatible system. However, the methodology applied is based on a mixture of both aqueous and organic solutions; this fact makes that during the fabrication process, a microemulsion of discrete droplets of the organic phase in a continuous aqueous phase is formed leading to segregation. Phase separation was reported for Ch/PCL blends obtained by casting; in this case the polysaccharide (10–30%) segregated in nano or microaggregates depending on the content and it appeared inside the PCL spherulites [103]. In other blends, no phase separation was formed in membranes with 25–75% PCL when using homogeneous solutions and dilute concentrations [96]. Returning to morphology of our Sr(II) membranes, the SEM images revealed agglomerates of the strontium crystals mainly on the surface of the continuous matrix; these agglomerates presented a morphology that resembles the “cauliflower” observed in the apatite-like layer usually formed upon immersion of sample in physiological body fluid (SBF). The exposition of the salt crystals on the surface will favor the availability of the Sr(II) ions to interact with the biological medium.

Structural characterization of Ch/2PCL and Ch/PCL samples reveals differences in some FTIR absorption bands compared to those of pure components. The broadness of band between 3500 and 3200 cm^−1^ indicates that during the cross-linking process some hydrogen bonds in the chitosan structure were destroyed and other new between the chitosan and TPP were formed [87]. In the region between 1750–1500 cm^−1^, the band due to υ C=O in ester groups shifts with respect to pure PCL as reported by García Cruz et al. for biodegradable porous Ch/PCL semi-interpenetrating polymer networks (semi-IPNs) [104]; the bands due to υ C=O (amide I) and to δ N–H in NH_3_^+^ groups shift compared to pure chitosan what can be attributed to ionic interactions between the protonated amino groups and the negatively charged phosphate groups, as it was reported for other chitosan membranes cross-linked with TPP [87,95]. But this shift has also been ascribed to hydrogen bond interactions between the natural polymer and PCL [104]. Finally, in the region between 1300–900 cm^−1^, the appearance of bands due to υ_as_ PO_2_ groups in TPP ions indicates the presence of the cross-linking agent [87]. Likewise, EDS spectra of Sr(II) membranes show the peaks of Sr and P coming from TPP. TGA evaluation of samples supports the formation of hybrid composite materials showing different thermal behavior than that of a simple mixture of pure components.

Water uptake is important for tissue engineering but particularly when the scaffold will perform in the oral cavity [105]. The studied membranes demonstrated their ability to absorb and retain a noticeable amount of water after immersion in PBS. However, some differences were observed between blank and Sr(II) samples. In the blanks the Ch:PCL ratio plays the main role in the sense that the higher the PCL content the lower the maximum *WU*, suggesting that water preferably absorbs into the hydrophilic polysaccharide. However, both compositions of Sr(II) membranes swell following a similar pattern and attain maxima *WU* values (≈245%) suggesting that the Sr(II) located on the surface is playing the dominant role. Morphology of Sr(II) membranes after 30 days soaking analyzed by SEM shows signs of erosion of the chitosan matrix as a consequence of water intrusion, scarce Sr(II) crystals and unaltered PCL microparticles attributed to the long-term degradability of the synthetic polymer [106]. The observed microstructure indicates that Sr(II) scaffolds have dimensional stability for the studied period and that most of the Sr(II) crystals have interchanged with the medium within this time.

The biocompatibility of biomaterials is closely related to cell-materials interactions and, in particular to cell adhesion to their surface. Attachment, adhesion and spreading of cells are the first step of these interactions and the quality of these processes will influence the cells capability to proliferate and to differentiate itself on contact with the scaffold [107]. In particular, cell adhesion and proliferation is highly important in biomaterials designed for tissue engineering purposes. Cell adhesion onto the studied membranes was examined by SEM using MG-63 and hBMSCs cell lines. Micrographs showed that both types of cells adhered, proliferated and formed ECM best onto the Sr(II) membranes compared to the blanks, what, additionally indicated that there was no direct toxic effects and cellular metabolism was normal. Additionally, the Sr/Ch/PCL composition distinguished with a higher cell adhesion and proliferation of MG-63 cells compared to the Sr/Ch/2PCL system, in the period from 1 to 6 days. Accordingly, several authors have been demonstrated that the presence of strontium in ceramics showed enhancing effects on osteoblasts cell growth and also ALP activity [108]. In further studies, the Sr/Ch/PCL system also demonstrated its capacity to adhere and grow hBMSCs, and cell population was much more abundant compared with the blank sample, especially at 7 days post seeding what can be attributed to the presence of Sr(II) ions. Cao et al. developed a modified chitosan/PCL electrospun fibrous scaffold charged with bone morphogenetic protein-2 (BMP-2) and found that MSCs attached readily with increasing spreading [109]. Similar observations were reported by composite chitosan scaffolds based on bactericide SrHAp/chitosan systems synthetized by Lei et al. [10] and composites of nSrHAp/chitosan developed by Xu et al. [76] produced favorable adhesion, spreading and proliferation of hBMSCs and enhanced osteoinductivity.

Quantitative analysis on cell proliferation after direct seeding of cells is usually assessed by AB assay [83]. Evaluation of our Sr(II) membranes using osteoblast-like cells and hBMSCs showed satisfactory cell viability after 21 days. Particularly, the Sr/Ch/PCL membrane highlighted by showing significant increase of MG-63 cells viability compared to the blank at 7 and 21 days. These findings seem to suggest again that both presence of the Sr(II) ions and the hydrophilic/hydrophobic balance of the sample can play an important role in the cell proliferation processes. Osteogenic response of our Sr/Ch/PCL membranes was investigated by DNA and ALP quantification. ALP activity is one of the characteristic parameters of osteoblast cells differentiation and a signal for the subsequent production of the proteins leading to mineralization [86,110]. In our studied Sr(II) membranes ALP content normalized for DNA was significantly higher compared to the blank in MG-63 cell culture at 14 days and in hBMSCs culture at 7 and 14 days. These results show the osteogenic capacity of the hybrid membranes that can be tentatively attributed to the presence of Sr(II) ions, although according to Seol et al., chitosan sponges can enhance ALP [51]. Generally, osteoconductive properties are attributed to the presence of inorganic components such as HAp, BG, or calcium phosphate salts. This behavior was also found by Kong et al. for chitosan/nHAp composite scaffolds using the MC 3T3-E1 preosteoblast cell line derived from newborn mouse calvaria [62] and by Shalumon et al. for nHAp and nBG incorporating PCL/chitosan composite scaffolds tested with human periodontal ligament fibroblast cells (hPLFs) and osteoblast like cells (MG-63 cell line) [111]. Both researchers found that the ALP activity of cells on the composite scaffolds increased compared with those of only chitosan, showing a higher differentiation level. Respect to the chitosan composite scaffolds with Sr(II), Su et al. have demonstrated that the strontium phosphate can affect the ALP activity in the osteogenic process when they test hydrogels formed with chitosan and strontium in presence of MSCs. The increase in the osteogenic expression, ALP activity, and calcium deposition indicates the effect of strontium in enhancing bone remodeling and bone structure stabilization [112]. Martín-del-Campo et al. demonstrated that the presence of Sr(II) ions stimulate hDPSCs proliferation, matrix mineralization and ALP activity [79]. More recently, Lei et al. observed that the release of Sr(II) ions from SrHAp/chitosan scaffolds enhanced ALP activity of hBMSCs and ECM mineralization [10].

In vivo biocompatibility was studied by implantation of Ch/PLC and Sr/Ch/PLC membranes using collagen (commercial collagen membrane material) as control. All systems where implanted in the backs of rats which were sacrificed at specific time points (7, 14 and 28 days) to observe the histological response using light microscopy. In a previous work where biocompatibility of cross-linked chitosan hydrogels was studied [113], non-degraded chitosan hydrogels were stained with eosin (pink) whereas degradable chitosan hydrogels were stained with the basophilic hematoxylin (blue). Azab et al. [113] proposed that the change in the staining patter was due to the shift in charge of the degraded chitosan from positive to negative. In our work, histological images (Figure 10 and Figure 11) showed chitosan membranes stained with eosin (pink). At physiological conditions chitosan is positively charged, which leads to its staining by negative charged eosin. In the in vivo subcutaneous biocompatible study in rats, all studied and control membranes exhibited inflammatory and tissue responses. This acute inflammatory response was somewhat expected in line with the typical reported host reactions following biomaterial implantation [114]. This kind of reaction has been reported even for nontoxic biomaterials such as silk, collagen or PLA [115]. The inflammatory reaction observed after subcutaneous implantation of the materials represented a typical acute response upon injury.

Regarding membrane degradation, in Bavariya et al. work [116], significant degradation of chitosan membranes was not evident in histological sections until 16–20 weeks post implantation as compared with significant degradation of collagen membrane at 12 weeks. In our work, collagen membranes appeared more disorganized along time, in contrast with the studied membranes, which were stable for all the experimental time. In some cases, histology images showed membrane fragments possible due to the characteristic chitosan erosion. Clinicians have suggested ideal membrane degradation time 4–6 months for large defects to provide sufficient time for bone regeneration [117,118].

Tissue inflammatory response to the two types of studied membranes was mild compared to the inflammatory response caused by the absorbable collagen control membrane. Collagen membranes showed signs of acute and chronic inflammation in the rat, mostly due to the presence of multinucleated giant cells surrounded fragments and rest of the membrane. The presence of membrane fragments evoked a more cellular reaction in comparison with stable studied membranes. However, after subcutaneous implantation of membranes of the three groups, no physiological signs of severe inflammation were observed. The studied materials induced a mild inflammatory infiltrate mainly composed by mononuclear cells, i.e., monocytes, macrophages, lymphocytes and some fibroblasts.

The extension of the observed inflammatory infiltrate was slightly higher in Ch/PLC group than in Sr/Ch/PLC one demonstrating that the resolution of the inflammatory process was faster for Sr/Ch/PLC membranes. The inflammatory response triggered by Ch/PLC membranes was often accompanied by abundant monocytes and macrophages population, and more extensive necrosis when compare with Sr/Ch/PLC membrane. The granulation tissue showed progressive increased replacement by fibrous connective tissue being less developed at Sr/Ch/PLC membranes than in Ch/PLC. The reaction to Sr/Ch/PLC membranes evoked less development of fibrosis, with a thinner fibrous tissue compared with Ch/PCL. The focal fibrous tissue was formed by the parallel deposit of few collagen fibers.

Based on the histological observation all types of membranes can therefore be considered biocompatible, highlighting the most favorable response to Sr/Ch/PCL system. Additionally, the studied membranes Ch/PLC and Sr/Ch/PCL maintain their structural integrity for 28 days period highly recommended for bone regeneration purposes, opposed to collagen membranes which disintegrated and appeared forming a mesh-like structure.

## 5. Conclusions

Sr(II) containing scaffolds consisting of a continuous matrix of TPP cross-linked chitosan and PCL microparticles were fabricated in a facile two-step casting/evaporation method using Ch:PCL ratios of 1:2 and 1:1. Both compositions presented good physico-chemical properties, swelling content around 250% and presented dimensional stability for at least one month. They did not show in vitro cytotoxicity against neither MG-63 cell line nor hBMSCs. The system Sr/Ch/PCL showed qualitatively and quantitatively higher cell proliferation, offering good support for adherence and proliferation of cells. Specifically, cellular studies on the Sr/Ch/PCL system using hBMSCs demonstrated a noticeable enhancement of DNA content and ALP activity as well as good and adequate niche for adherence and proliferation of cells. In vivo experiments in rats manifested good biocompatibility for all studied membranes standing out the results obtained for the Sr/Ch/PCL system. All these results allow considering this Sr(II) hybrid system as a promising biomaterial for application of bioactive scaffolds in bone tissue engineering such as the treatment of craniofacial regeneration as well as on the active regeneration of other bone tissues, considering that the developed formulation acts as a well-defined route of local delivery of Sr(II) ions. In addition, the in vivo response of the implanted systems did not apparently show signs of toxicity avoiding the negative secondary effects described after administration of strontium by systemic route.

## Figures and Tables

**Table 1:** Names and compositions of blank and Sr(II) containing membrane scaffolds.

Name	Code	Ch/PCL (*wt*/*wt*)	SrF_2_ (wt % Respect to Ch)
Blank membranes	Ch/2PCL	1:2	-
	Ch/PCL	1:1	-
Sr(II) membranes	Sr/Ch/2PCL	1:2	5
	Sr/Ch/PCL	1:1	5

Ch: chitosan; PCL: poly(ε-caprolacone).

## Supplementary Materials

The following are available online at http://www.mdpi.com/2073-4360/10/3/279/s1, Figure S1: ATR-FTIR spectra of pure Ch and PCL and Ch/2PCL and Ch/PCL membranes, Figure S2: TGA (Termogravimetric analysis) (a) and DTG (Derivative Thermogravimetric analysis). (down) of pure Ch and PCL, Figure S3: SEM images of Sr(II) membranes after 30 d immersion in PBS buffer at 37 °C. a) 200x; b) 500x; c) 1000x, Figure S4: In vitro cytotoxicity results of blank and Sr(II) membranes using MG-63 cells (a) and hBMSCs (b). Results are given as mean ± sd (*n* = 5), Table S1: DTG (Derivative Thermogravimetric analysis) results of pure PCL (poly(ε-caprolactone), chitosan (Ch), blank and Sr(II) membranes.
