# Supplementary material for: Bioactive Sr(II)/Chitosan/Poly(ε-caprolactone) Scaffolds for Craniofacial Tissue Regeneration. In Vitro and In Vivo Behavior
Source: Polymers (Basel). 2018 Mar 7;10(3):279. doi: 10.3390/polym10030279 (PMC6415099; doi:10.3390/polym10030279)
Supplement: Supplementary file 1 [file polymers-10-00279-s001.pdf]

# Supporting Information

## Bioactive Sr(II) loaded chitosan/PCL scaffolds for bone tissue regeneration. Biphasic scaffolds for the bioactive regeneration of bone tissue

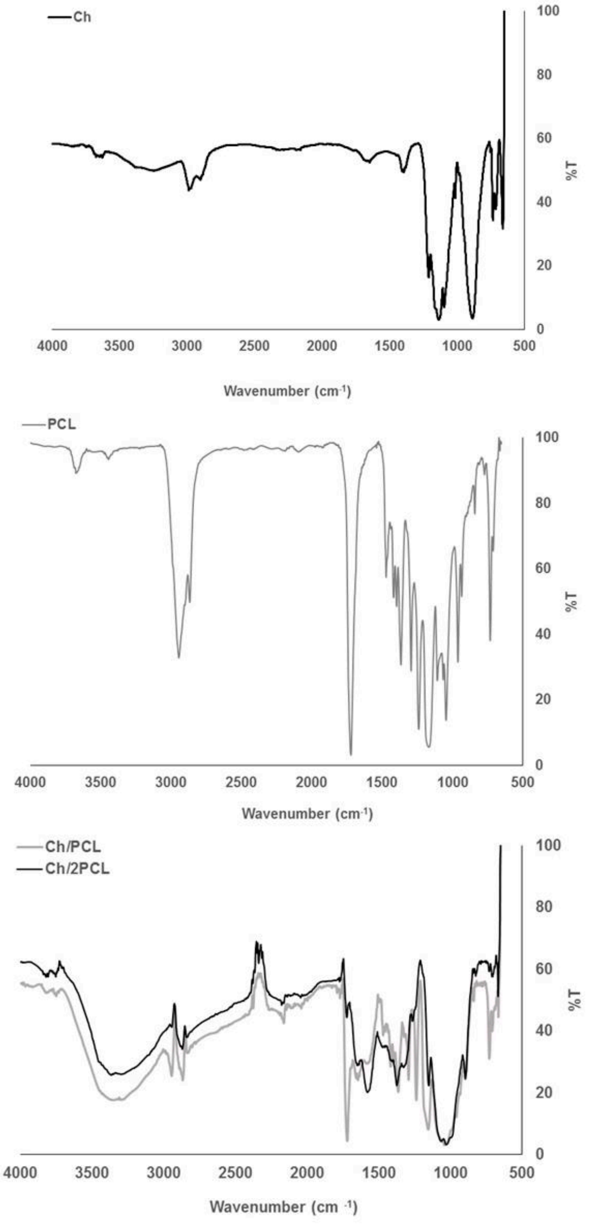


**Figure S1.** ATR-FTIR spectra of pure Ch and PCL and Ch/2PCL and Ch/PCL membranes. Ch: chitosan; PCL: poly(ε-caprolactone).

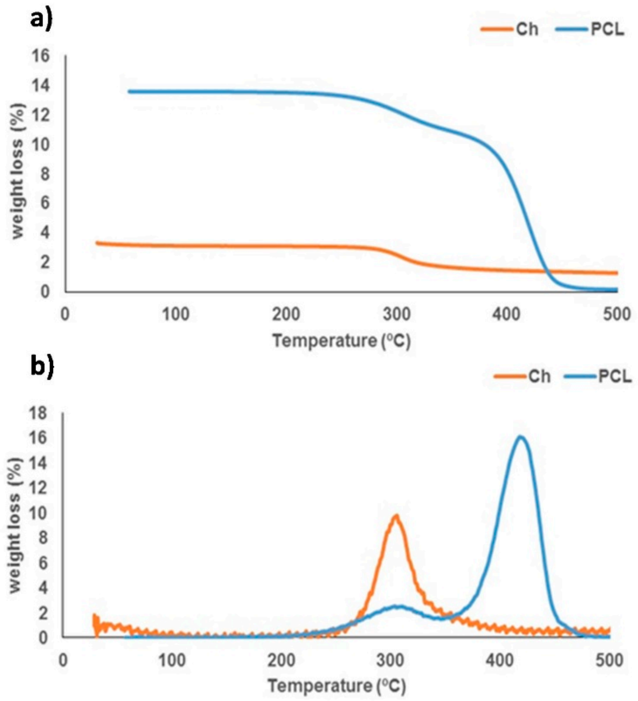


**Figure S2.** TGA (Termogravimetric analysis) (a) and DTG (Derivative Thermogravimetric analysis). (down) of pure Ch and PCL. Ch: chitosan; PCL: poly(ε-caprolactone).

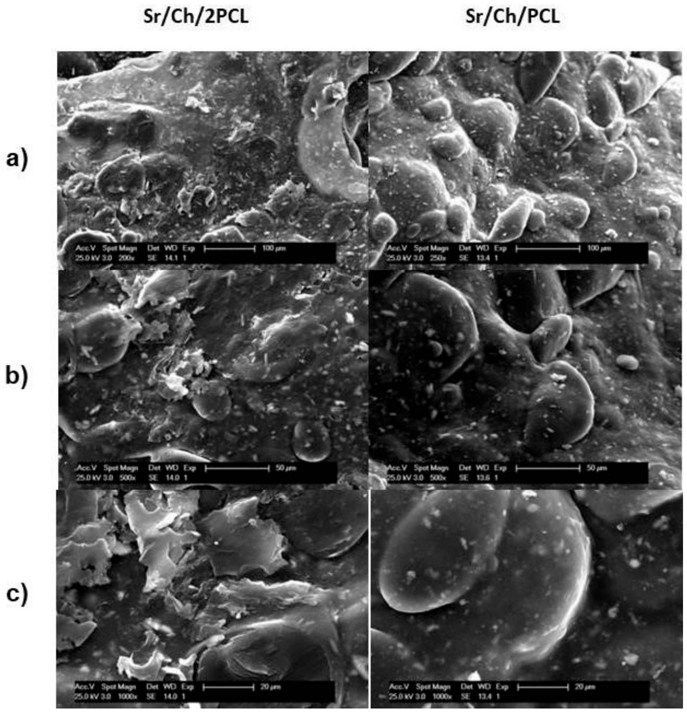


**Figure S3.** SEM images of Sr(II) membranes after 30 d immersion in PBS buffer at 37 °C. a) 200x; b) 500x; c) 1000x. Ch: chitosan; PCL: poly(ε-caprolactone).

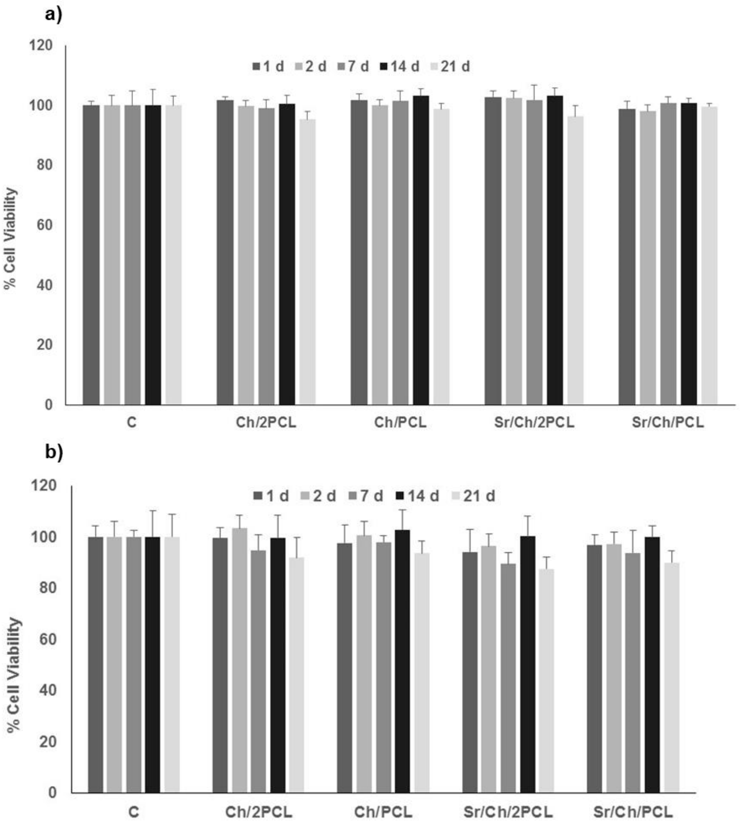


**Figure S4.** In vitro cytotoxicity results of blank and Sr (II) membranes using MG-63 cells (a) and hBMSCs (b). Results are given as mean ± sd (*n* = 5). Ch: chitosan; PCL: poly(ε-caprolactone).

**Table S1.** DTG (Derivative Thermogravimetric analysis) results of pure PCL (poly(ε-caprolactone), chitosan (Ch), blank and Sr(II) membranes.

| Sample | $T_{max1}$ (°C) | $T_{max2}$ (°C) |
|---|---|---|
| Ch | 287 | - |
| PCL | 307 | 419 |
| Ch/2PCL | 271 | 416 |
| Ch/PCL | 290 | 420 |
| Sr/Ch/2PCL | 282 | 410 |
| Sr/Ch/PCL | 286 | 418 |
